# Effects of *Allium victorialis* leaf extracts and its single compounds on aldose reductase, advanced glycation end products and TGF-β1 expression in mesangial cells

**DOI:** 10.1186/1472-6882-13-251

**Published:** 2013-10-03

**Authors:** Young Sook Kim, Dong Ho Jung, Ik Soo Lee, So-Jin Choi, Song Yi Yu, Sea-Kwang Ku, Myung-Hwa Kim, Jin Sook Kim

**Affiliations:** 1 Korean Medicine-Based Herbal Drug Development Group, Herbal Medicine Research Division, Korea Institute of Oriental Medicine (KIOM), Daejeon, Republic of Korea; 2 Developmnent Team for the New Drug of Oriental Medicine, Daegu Haany University, Gyeongsan, Republic of Korea; 3 Jeil Pharmaceutical CO., LTD, Yongin, Kyonggi-do, Republic of Korea; 4 Present address: Korea Drug Development Fund, 21-1 Migeun-dong, Seodaemun-gu, Seoul 120-020, Republic of Korea

**Keywords:** *Allium victorialis*, Aldose reductase, Advanced glycation end products, Diabetic nephropathy, transforming growth factor-beta1, Mouse mesangial cells

## Abstract

**Background:**

Accumulating evidences suggest that aldose reductase (AR) inhibitors and advanced glycation end product (AGE) formation inhibitors may prevent chronic hyperglycemia-induced long-term complication in diabetes. Transforming growth factor-beta1 (TGF-β1) plays an important role in the development of diabetic nephropathy. *Allium* species have been utilized in folk medicine throughout the world for the treatment of various physical disorders. However, the benefits of *Allium victorialis* (*A. victorialis*) against diabetic complications, especially nephropathy, have yet to be explored. In the present study, we investigated the protective effect of the compounds isolated from *A. victorialis* leaf on diabetic nephropathy.

**Methods:**

*In vitro* AR activity, AGEs formation, and AGE-receptor for AGEs (RAGE) binding in human RAGE (hRAGE)-overexpressing cells were tested. High glucose-induced transforming growth factor-beta1 (TGF-β1) expression was also examined in mouse kidney mesangial cells (MMCs) cultured under high glucose.

**Results:**

Of the isolated eight compounds from *A. victorialis* leaf extracts tested, quercitrin exhibited the most pronounced inhibitory effects on AR activity (IC_50_ value of 0.17 μM) and AGEs formation (IC_50_ value of 4.20 μM). Furthermore, quercitrin disrupted AGE-RAGE binding in a concentration-dependent manner in hRAGE-overexpressing cells. Additionally, of the eight compounds tested, ferulic acid significantly reduced high glucose-induced TGF-β1 expression and secretion in MMCs.

**Conclusions:**

Our results suggest that active compounds isolated from *A. victorialis* leaf exhibit inhibitory effects on AR activity in rat lenses and AGE formation. Further, ferulic acid reduces TGF-β1 mRNA expression and secretion in MMCs under diabetic conditions. Thus, *A. victorialis* is a good candidate for the development of treatments for diabetic nephropathy.

## Background

Chronic hyperglycemia is the most common feature of all forms of diabetes mellitus, and it accelerates the induction of aldose reductase (AR, EC 1.1.1.21) and the irreversible formation of advanced glycation end products (AGEs), which play important roles in the pathogenesis of diabetic complications [1]. Diabetic nephropathy is a major complication of diabetes mellitus, and although the mechanism of glomerulosclerosis still remains unclear, the irreversible formation of AGEs, polyol accumulation, and oxidative stress have been considered the major causes of diabetic nephropathy [2]. AR, the first rate-limiting enzyme in the polyol pathway, is present in the eyes, kidneys, and other tissues affected by diabetic complications. Increased glucose enters the polyol pathway, where it is reduced by AR to sorbitol [2,3]. AR inhibitors (ARIs), such as epalrestat, 3,3-tetramethyleneglutaric acid (TMG), and fidarestat, have been developed, and some have been revealed to prevent diabetic nephropathy in animal models or patients [3-7]. ARIs from natural products have been found to prevent or delay the development of diabetic complications in animal models [8-10].

Transforming growth factor-beta 1 (TGF-β1) is a multifunctional cytokine that plays important roles in cell proliferation, wound healing, differentiation, apoptosis, and the immune response in several cells [11]. In particular, TGF-β1 is a key mediator of diabetic nephropathy that increases the levels of extracellular matrix (ECM) proteins, such as collagen I and IV, laminin, and fibronectin, in the glomeruli [11]. In addition, TGF-β has been identified as a critical regulator and mediator of pathophysiological processes of ocular tissue development or repair. TGF-β–mediated signaling is involved in the progression of diabetic nephropathy, and high levels of TGF-β are found in diabetic kidneys.

Natural products and their active constituents have been reportedly used for the treatment of diabetes and diabetic complications [10]. The genus *Allium* comprises more than 600 different species distributed throughout North America, North Africa, Europe, and Asia. Many *Allium* species have been utilized in folk medicine throughout the world for the treatment of various physical disorders such as burns, wounds, headaches, chest colds, and rheumatism [12]. *Allium victorialis* var. *platyphyllum* (Liliaceae), one of the most popular *Allium* species, is an edible perennial herb widely distributed on Ulleung Island and Mt. Hambeak of the Korean Peninsula. Recently, *Allium victorialis* (*A. victorialis*) has received much attention owing to its diverse and potentially significant pharmacological properties including antiarteriosclerotic, anticancer, antioxidant, antidiabetic, antiobesity, antineuroinflammatory, hepatoprotective, and nephroprotective effects [12-21].

In this paper, we examined the effects of eight compounds (**1**–**8**) isolated from *A. victorialis* leaf on AR activity, AGE formation, and TGF-β1 mRNA expression and protein secretion in mouse glomerular mesangial cells (MMCs) cultured under diabetic conditions. Furthermore, binding between AGE and receptor for AGE (RAGE) in human RAGE (hRAGE)-overexpressing MMCs was analyzed, and the most active compound was identified. These results show that single compounds from *A. victorialis* leaf extracts have preventive effects against diabetic nephropathy and may be useful as candidates for preclinical study in the treatment of diabetic nephropathy.

## Methods

### Plant materials and chemicals

The leaf of *A. victorialis* were purchased from a commercial supplier in Goryung, (Gyeongbuk, Korea, in January, 2005) and identified by Prof. K-R Park in the Department of Herbology, The Medical Research center for Globalization of Herbal Formulation, Daegu Haany University. A herbarium voucher specimen (no. KIOM-ALVI) has been deposited at the Herbarium of the Diabetic Complications Research Group, Korea Institute of Oriental Medicine. Antibodies were purchased from Cell Signaling (Beverly, MA) and Santa Cruz Biotechnology (Santa Cruz, CA). All other reagents were obtained from Sigma-Aldrich (St. Louis, MO). Reagents used for cell culture were purchased from GIBCO-BRL (Grand Island, NY).

### General experimental procedures

Optical rotations were measured on a JASCO P-2000 digital polarimeter. Hydrogen 1 (300 MHz) and carbon 13 nuclear magnetic resonance (NMR; 75 MHz) spectra were obtained using a Bruker DRX-300 spectrometer with tetramethylsilane as an internal standard. Two-dimensional-NMR experiments (correlation spectroscopy, heteronuclear multiple-quantum correlation, and heteronulear multiple bond correlation) were run on a Bruker Avance 500 NMR spectrometer. Electrospray ionization mass spectrometry spectra were recorded on a Shimadzu liquid chromatography-mass spectrometry-ion trap-time of flight spectrometer. Column chromatography was performed using silica gel (70–230 mesh, Merck), YMC-gel ODS-A (12 nm, S-75 μm, YMC), and Sephadex LH-20 (Amersham Pharmacia Biotech). Thin-layer chromatography was performed on pre-coated silica gel 60 F_254_ (0.25 mm, Merck) and RP-18 F_254s_ plates (0.25 mm, Merck). Spots were detected by utraviolet light (254 nm) and spraying with 10% H_2_SO_4_ followed by heating.

### Extraction and isolation

The air-dried leaf of *A. victorialis* (4.0 kg) were extracted with 50% EtOH (36 L) at 60°C for 5 h, filtered, and concentrated to yield a 50% EtOH extract (1.0 kg). This extract (1.0 kg) was suspended in H_2_O (4 L) and then partitioned successively with EtOAc (3 × 4.0 L) and *n*-BuOH (3 × 4.0 L) to afford EtOAc- (13 g) and *n*-BuOH-soluble fractions (258 g), respectively. The EtOAc- (12 g) and *n*-BuOH-soluble fractions (250 g) were subjected to a series of chromatographic techniques including silica gel, YMC RP-18, and Sephadex LH-20 column chromatographies, leading to the isolation of eight compounds (**1**-**8**, Table 1), Kaempferol 3,7,4’-O-β-D-triglucopyranoside (**1**, 280 mg), Kaempferol 3,7-O-β-D-diglucopyranoside (**2**, 66 mg), kaempferol 3,4’-O-β-D-diglucopyranoside (**3**, 70 mg), quercitrin (**4**, 10 mg), kaempferol (**5**, 24 mg), quercetin (**6**, 45 mg), 4-hydroxycinnamic acid (**7**, 4.3 mg), and ferulic acid (**8**, 10 mg).

**Table 1:** **Inhibitory effect of extracts, fractions, and compounds isolated from *A. victorialis *** **on AR and AGEs formation**

**No.**	**Extracts, fractions, and isolated compounds**	**Mw**	**AR IC**_**50**_	**AGEs IC**_**50**_
1	Kaempferol 3,7,4’-O-β-D-triglucopyranoside	772.66	>50 μM	>100 μM
2	Kaempferol 3,7-O-β-D-diglucopyranoside	610.52	>50 μM	56.44±1.39 μM
3	Kaempferol 3,4’-O-β-D-diglucopyranoside	610.52	9.77±0.33 μM	59.66±1.22 μM
4	Quercitrin	448.38	0.17±0.10 μM	4.20±0.04 μM
5	Kaempferol	286.24	1.10±0.63 μM	36.01±1.40 μM
6	Quercetin	302.24	3.61±0.19 μM	27.10±0.11 μM
7	4-Hydroxycinnamic acid	164.16	>50 μM	9.92±0.11 μM
8	Ferulic acid	194.16	>50 μM	7.50±0.20 μM
9	*A. victorialis* 50% EtOH		>10 μg/ml	>75 μg/ml
10	*A. victorialis* EtOAc		7.53±0.02 μg/ml	30.13±1.68 μg/ml
11	*A. victorialis* BuOH		>10 μg/ml	>75 μg/ml
12	Tetramethyleneglutaric acid	186.20	5.07±0.06 μM (0.94±0.01 μg/ml)	-
13	Aminoguanidine	74.1	-	1.03±0.07 mM (76.47±4.81 μg/ml)

### Rat lens AR activity

AR activity was measured as described previously [9,22]. All animal procedures were approved by the Korea Institute of Oriental Medicine Institutional Animal Care Committee on animal care at our institute and conducted according to institutional guidelines. Rat lenses were isolated from the eyes of 8-week-old Sprague–Dawley rats (Orient Co., Seongnam, Korea) and homogenized in 12 volumes of 150 mM sodium phosphate buffer (pH 6.2) and 10 mM 2-mercaptoethanol. The homogenate was centrifuged at 14,000 rpm for 30 min, and the supernatant was used as crude rat lens AR. The incubation mixture contained 150 mM sodium phosphate buffer, 0.15 mM nicotinamide adenine dinucleotide phosphate (NADPH), 10 mM dl-glyceraldehyde as a substrate, and 700 μg/ml of enzyme substrate, with or without compounds or positive control, in a total volume of 1.0 ml. The reaction was initiated by the addition of NADPH at 37°C and stopped by the addition of 0.15 ml of 0.5 N HCl. Next, 0.5 ml of 6 M NaOH containing 10 mM imidazole was added, and the solution was heated at 60°C for 15 min to convert NADP to a fluorescent product. The fluorescence (ex. 360 nm/ em. 460 nm) was assayed using a spectrofluorometric detector (Synergy HT, Bio-Tek, Winooski, VT). The concentration of each test sample that inhibited activity by 50% (IC_50_) was estimated from the least-squares regression line of the logarithmic concentration plotted against the remaining activity.

### Determination of AGEs formation

AGEs formation assay was performed as previously described [23,24]. Bovine serum albumin (BSA, 10 mg/ml, Sigma-Aldrich) in 50mM phosphate buffer (pH 7.4) with containing 0.02% sodium azide to prevent bacterial growth was added to 0.2 M fructose and glucose. The reaction mixture was then mixed with compounds or aminoguanidine (AG, Sigma-Aldrich). After incubating at 37°C for 7 days, the fluorescent reaction products were assayed on a spectrofluorometric detector (BIO-TEK, Synergy HT, Ex: 350 nm/Em: 450 nm). AGEs assay was performed in quadruplicate. The concentration of each test sample giving 50% inhibition of the activities (IC_50_) was estimated from the least-squares regression line of the logarithmic concentration plotted against the remaining activity.

### Cell Cultures

Mouse kidney mesangial cells (SV40 MES13, MMC) were obtained from the American Type Culture Collection (#CRL-1927, Rockville, MD) and cultured in Dulbecco's modified Eagle's medium:F-12 (3:1) supplemented with 14 mM HEPES, penicillin 100 U/ml, streptomycin 100 μg/ml, and 5% fetal bovine serum. Cells were routinely grown to confluence in a humidified 37°C, 5% CO_2_ incubator.

### RNA extraction and semi-quantitative reverse transcription-polymerase chain reaction (RT-PCR) analysis

Total cellular RNA was extracted with TRIzol (Invitrogen, Carlsbad, CA), quantified by measuring the absorbance at 260 nm, and stored at -80°C until analysis. The expression of TGF-β1 and GAPDH mRNAs was detected by RT-PCR analysis. The extracted RNA (1 μg) was subjected to a reverse transcriptase reaction with the Maxime RT premix (Intron, Daejeon, Korea) at 42°C for 60 min and 72°C for 10 min. Subsequently, semi-quantitative PCR was performed with Accupower® PCR premix (Intron, Daejeon, Korea). The primer sequences were as follows: mouse TGF-β1 (sense) 5’- TGA ACC AAG GAG ACG GAA TAC AGG -3’, (anti-sense) 5'- GCC ATG AGG AGC AGG AAG GG -3’ and mouse GAPDH (sense) 5’- ACG GCA AAT TCA ACG GCA CAG -3’, (anti-sense) 5’- AGA CTC CAC GAC ATA CTC AGC AC -3’. Aliquots of PCR products were electrophoresed on 1.2% agarose gels and visualized after ethidium bromide staining.

### Determination of secreted TGF-β1 expression in MMCs using enzyme-linked immunosorbent assay (ELISA)

The levels of TGF-β1 in the medium were determined as described previously [9]. The medium was replaced with serum-free medium containing compound under high glucose conditions for 24 h. This medium was then harvested and TGF-β1 was activated by treatment with 1 N HCl (0.1 ml/0.5 ml of conditioned media) for 10 min at room temperature, then 0.1 ml 1.2 N NaOH/0.5 M HEPES was added. Quantikine mouse TGF-β1 ELISA (R&D systems, Minneapolis, MN) was performed according to the manufacturer’s protocol, and the TGF-β1 levels were normalized to those of total protein. Medium without cells that had been incubated under the same conditions was used as a control for the ELISA.

### Detection of live cell-based AGE-BSA/RAGE binding

AGE-BSA/RAGE binding in the cells was determined as described previously [23]. Briefly, Alexa 488 labeling of AGE-BSA was performed using the Alexa Fluor® 488 protein labeling kit (Molecular Probes, Eugene, OR). For the binding assay, human RAGE-overexpressing cells (1×10^4^) were seeded onto a 96-well assay plate with a clear bottom lid and black plate (Corning, NY) and incubated with serum-free media for 24 h. Before binding, 3% BSA was added for 30 min to block non-specific binding. Cells were treated with 5 μg of Alexa Fluor 488-labeled AGE-BSA in a total volume of 100 μl serum-free medium and incubated in the dark for 6 h in a 5% CO_2_ humidified atmosphere at 37°C. Compounds were added after the addition of AGE-BSA-Alexa Fluor 488 to hRAGE-overexpressing cells. The non-specific binding of AGE-BSA-Alexa Fluor 488 to cell surface proteins other than hRAGE was compared by incubating cells with untreated cells (blank). After binding, 100 μl Opti-MEM were added to the washed plates, and the plates were then analyzed using a microtiter plate reader (Bio-Tek, Winooski, VT) with excitation and emission wavelengths of 485 and 528 nm, respectively.

### Statistical analysis

Data are expressed as mean ± S.E.M. of multiple experiments. Paired Student's *t*-tests were used to compare two groups, or analysis of variance with Tukey’s was used for multiple comparison tests using PRISM software (Graph Pad, San Diego, CA). Values of *p* < 0.05 were considered statistically significant.

## Results and discussion

### Structure elucidation of compounds

**Figure 1:**
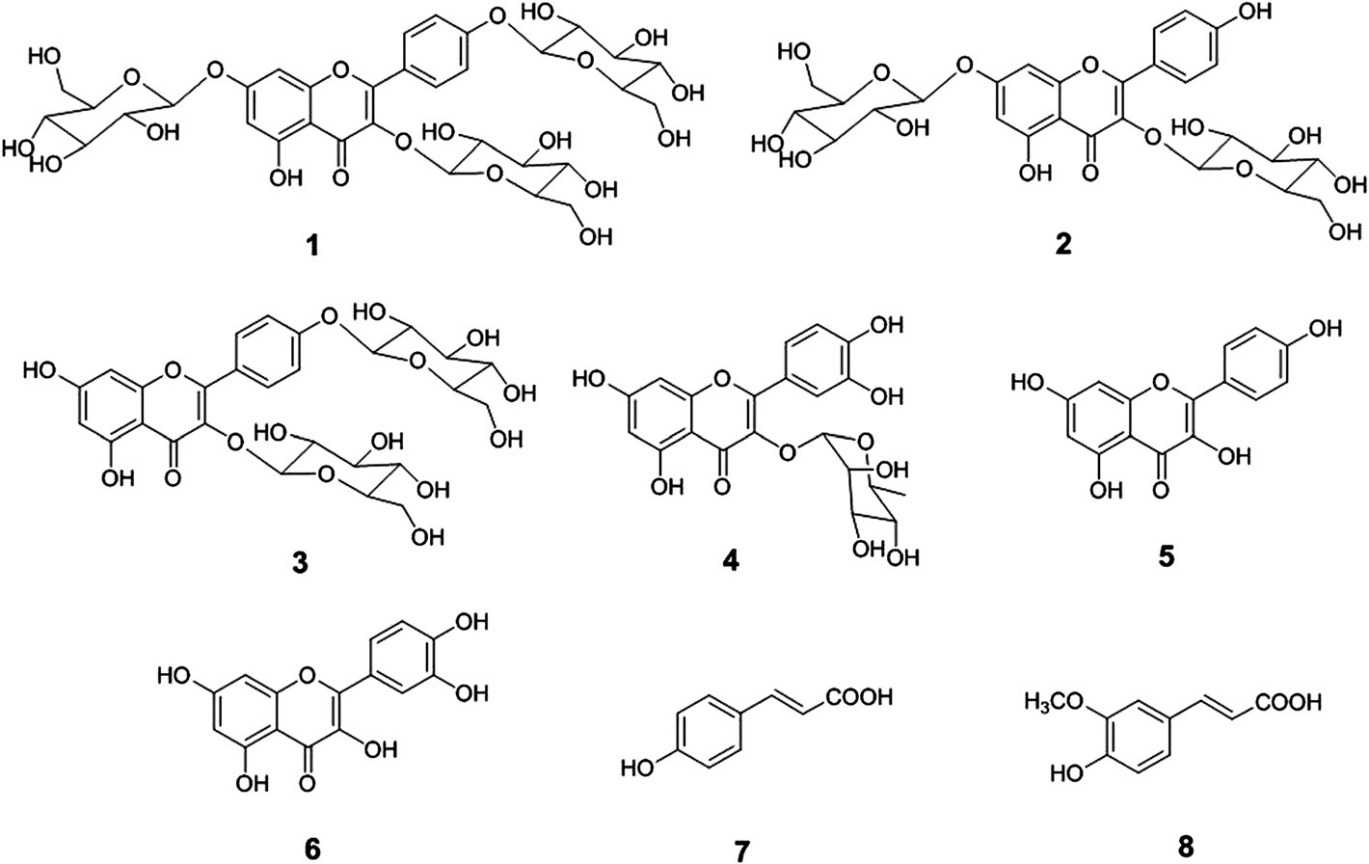
**Structures of the compounds (1–8) isolated from the leaf of *A. victorialis*.**

The EtOAc- and *n*-BuOH–soluble fractions were subjected to a series of chromatographic techniques, leading to the isolation of eight known compounds (**1**–**8**) (Figure 1). These compounds were identified as kaempferol 3,7,4′-*O*-β-d-triglucopyranoside (**1**), kaempferol 3,7-*O*-β-d-diglucopyranoside (**2**), kaempferol 3,4'-*O*-β-d-diglucopyranoside (**3**), quercitrin (**4**), kaempferol (**5**), quercetin (**6**), 4-hydroxycinnamic acid (**7**), and ferulic acid (**8**) by comparing their physicochemical and spectral data to those in the literature [25-31].

### Rat lens AR activity, AGE formation, and AGE/RAGE-binding in hRAGE-overexpressing cells

**Figure 2:**
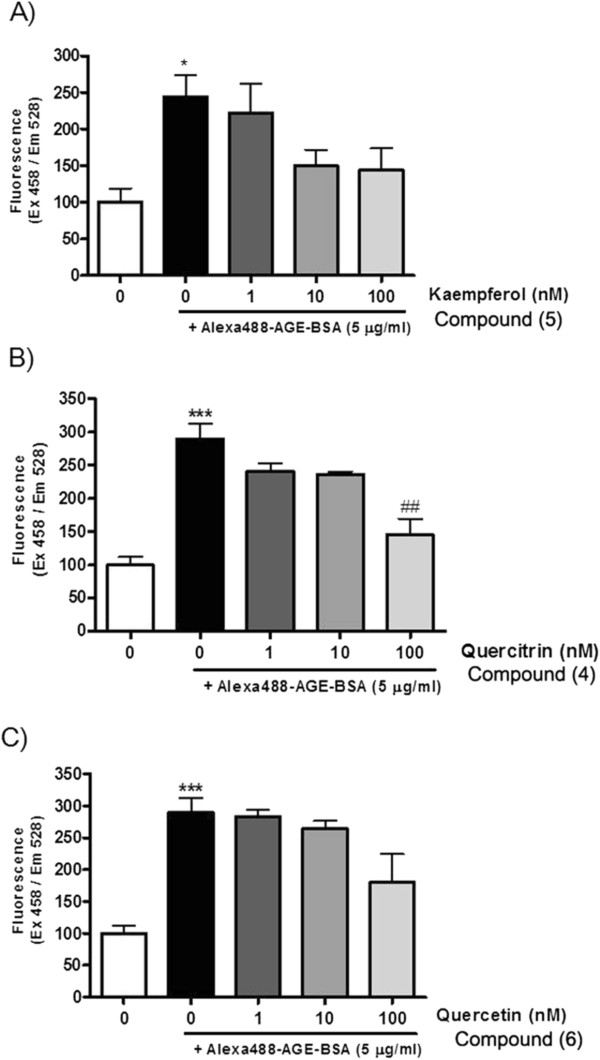
**Inhibitory effects of the isolated compounds on AGE-BSA/hRAGE binding in hRAGEoverexpressing MMCs.** Effects of the compounds (**A**. Kaempferol (5); **B**. Quercitrin (4); **C**. Quercetin (6)) on AGE/hRAGE binding were quantified by a fluorescence detector. Data are presented as the mean ± standard error of the mean (S.E.M.; n=4). ***p<0.001 vs. untreated cells; ##p<0.01 vs. cells treated with only Alexa Fluor 488- labeled AGE-BSA.

ARIs suppressing the hyperglycemia-induced polyol pathway have been identified as potential therapeutic candidates in the treatment and prevention of diabetic complications. The IC_50_ values of compounds (Table 1) in this assay were comparable to that those of known ARIs, such as TMG, which suggested that the compounds and extracts appeared to have an inhibitory effect on AR activity. Among the compounds, quercitrin (**4**), kaempferol (**5**), and quercetin (**6**) were significantly more potent than the previously known positive control, TMG. Previous research also demonstrated that flavonoids such as quercetin and myricitrin are effective inhibitors of lens AR [28]. We previously reported that quercitrin gallate also inhibits AR activity and xylose-induced lens opacity and oxidation [25]. Kaempferol and its prenylated derivatives are reported to be aldolase inhibitor [32]. Kaempferol 3,4’-O-β-d-diglucopyranoside (**3**) (IC_50_ = 9.77 ± 0.33 μM) and the *A. victorialis* EtOAc- soluble fraction (IC_50_ = 7.53 ± 0.02 μg/ml) inhibited AR activity. Although, IC_50_ level of EtOAc- soluble fraction was higher than TMG (0.94±0.01 μg/ml), among the extracts, it has the inhibitory effects on AGEs formation (IC_50_ =30.13±1.68 μg/ml; AG, IC_50_ = 76.47±4.81 μg/ml). Previous research indicated that genistein has inhibitory effects of AR activity in vitro, AGEs formation, and AGE-RAGE binding in hRAGE-overexpressing cells [9,23]. Next, we examined the inhibitory effects of compounds and extracts on AGEs formation (Table 1). Quercitrin (**4**) (IC_50_ = 4.20 ± 0.04 μM) and ferulic acid (**8**) (IC_50_ = 7.50 ± 0.20 μM) exhibited inhibitory effects on AGEs formation. Furthermore, because of the pronounced inhibitory effect of the three compounds (**4**, **5**, and **6)** on AR and AGEs formation, AGE-RAGE binding assays were performed in hRAGE-overexpressing cells (Figure 2). Among the compounds, quercitrin (**4**) significantly inhibited AGE-RAGE binding in hRAGE-overexpressing cells. Although quercitrin (**4**) has been tested on ARI effect [28], this compound has never been examined for the AGE-RAGE binding assay in hRAGE-overexpressing cells up to data. Quercitrin has anti-inflammatory effect through the inhibition of the NF-kappa B pathway and it shows potential anti-cancer effect, including cell cycle regulation and tyrosine kinase inhibition [33,34].

### Inhibition of high glucose-induced TGF-β1 expression and secretion in MMCs

**Figure 3:**
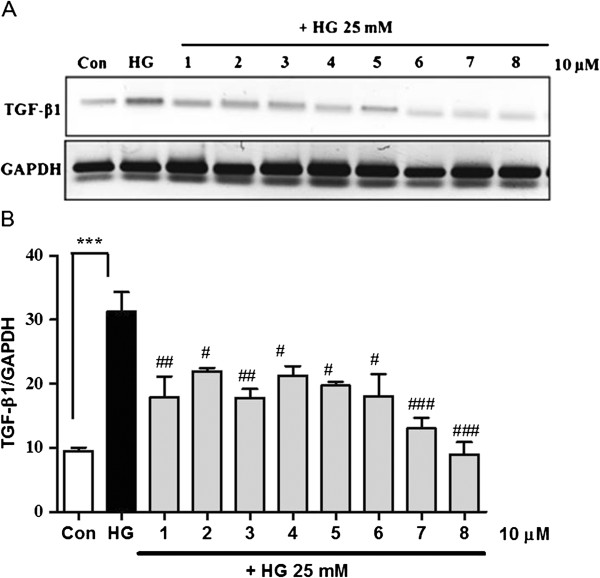
**Inhibitory effect of the compounds isolated from *A. victorialis *on high glucose-induced TGF-β1 mRNA expression.** Quiescent MMCs were treated with high glucose for 48 h followed by 10 μM of the compounds (**1–8)**. Kaempferol 3,7,4′-*O*-β-d-triglucopyranoside (**1**), kaempferol 3,7-*O*-β-d-diglucopyranoside (**2**), kaempferol 3,4'-*O*-β-d-diglucopyranoside (**3**), quercitrin (**4**), kaempferol (**5**), quercetin (**6**), 4-hydroxycinnamic acid (**7**), and ferulic acid (**8**). TGF-β1 mRNA expression was determined by relative RT-PCR. Results are the mean ± S.E.M. (*n*=3). ***p<0.001 vs. untreated cells; ###p<0.001, ##p<0.01, #p<0.05 vs. HG.

**Figure 4:**
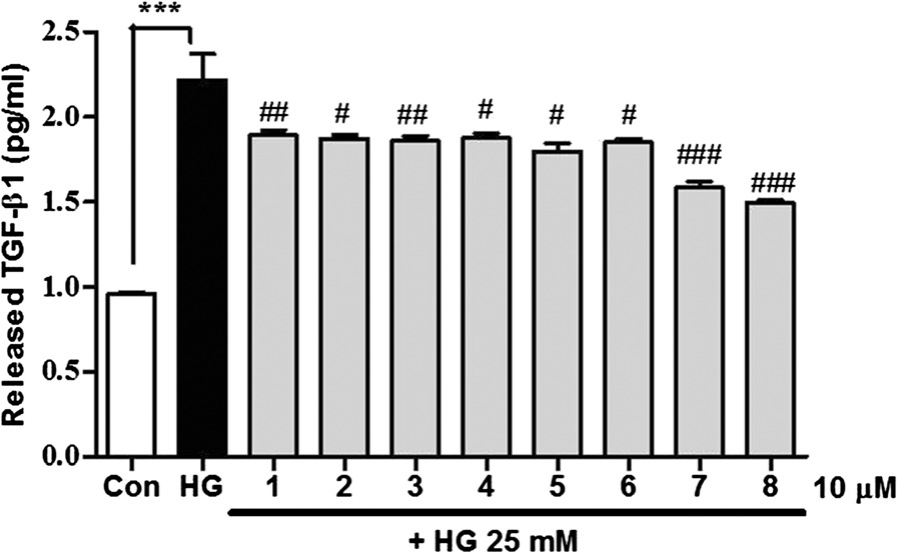
**Inhibitory effect of the compounds isolated from *A. victorialis *on high glucose-induced TGF-β1 secretion.** Quiescent MMCs were treated with high glucose for 48 h followed by 10 μM of the compounds (**1–8)**. To determine the amount of secreted TGF-β1 protein in the medium, ELISAs were performed on MMCs treated with high glucose. Experiments were done in triplicate on three separate occasions. Results are the mean ± S.E.M. (*n*=4). ***p<0.001 vs. untreated cells; ###p<0.001, ##p<0.01, #p<0.05 vs. HG.

TGF-β1 stimulates the production of ECM proteins such as fibronectin and collagen and promotes mesangial cell expansion [35,36]. In diabetic nephropathy, these changes are associated with the development of basement membrane thickening in the glomeruli [37]. Thus, TGF-β1 is considered a potential therapeutic target in diabetic nephropathy and other chronic renal diseases. To assess which compounds from *A. victorialis* are involved in the regulation of both TGF-β1 mRNA and protein levels in MMCs under diabetic conditions, cells were treated with high glucose in the presence or absence of single compounds (**1**–**8**) for 48 h. As shown in Figure 3A and B, single compounds (**1**–**8**) inhibited TGF-β1 mRNA expression in high glucose-stimulated MMCs (***p<0.001, vs. C; ###p<0.001, ##p<0.01, #p<0.05 vs. HG). Furthermore, we demonstrated that compounds from *A. victorialis* inhibit high glucose-induced TGF-β1 secretion (Figure 4, ***p < 0.001, vs. C; ###p<0.001, ##p<0.01, #p<0.05 vs. HG). Among the eight compounds identified from *A. victorialis*, ferulic acid (**8**) displayed the greatest inhibitory effect on TGF-β1 expression in MMCs. A previous study suggested that ferulic acid have protective effects against diabetic nephropathy by reducing oxidative stress and inflammation in a rat model of type 2 diabetes [38]. In the present study, we first demonstrated that the treatment of MMCs with single compounds from *A. victorialis* inhibited high glucose-induced TGF-β1 mRNA expression. However, toxicology study *in vivo* is needed to evaluate the safety of *A. victorialis* in the drug development.

## Conclusion

In summary, our data suggest that active compounds isolated from *A. victorialis* leaf exhibit inhibitory effects on AR activity and AGE formation. Further, ferulic acid reduces TGF-β1 mRNA expression and secretion in MMCs under diabetic conditions. Thus, the compounds isolated from *A. victorialis* leaf provide some scientific evidence to support the folk medicinal utilization of *A. victorialis* in the treatment of diabetic nephropathy. Furthermore, *A. victorialis* is a good candidate for the development of treatments for diabetic nephropathy.

## Competing interests

The authors declare that they have no competing interests.

## Authors’ contributions

YSK and JSK: Designed the study and wrote the manuscript; DHJ: Carried out the RAGE-AGE binding assay and cell culture experiments; SJC: Carried out the AGE and AR assays; ISL and SYY: Carried out the isolation of compounds; ISL: Helped to write draft the manuscript; SKK, MHK and JSK: Helped to discuss and supervised the work. All authors read and approved the final manuscript.

## Pre-publication history

The pre-publication history for this paper can be accessed here:

http://www.biomedcentral.com/1472-6882/13/251/prepub
